# StEPF2 and StEPFL9 Play Opposing Roles in Regulating Stomatal Development and Drought Tolerance in Potato (*Solanum tuberosum* L.)

**DOI:** 10.3390/ijms251910738

**Published:** 2024-10-05

**Authors:** Le Kang, Junke Liu, Hongqing Zhu, Leqin Liao, Muying Ye, Yun Wei, Nairong Liu, Qingbo Ke, Ho Soo Kim, Sang-Soo Kwak, Quanlu Zhou

**Affiliations:** 1 Key Laboratory of Nanchong City of Ecological Environment Protection and Pollution Prevention in Jialing River Basin, College of Environmental Science and Engineering, China West Normal University, Nanchong 637002, China; 2 Plant Systems Engineering Research Center, Korea Research Institute of Bioscience and Biotechnology, Daejeon 34141, Republic of Korea; 3 Institute of Soil and Water Conservation, Northwest A&F University, Yangling 712100, China; 4 Sweetpotato Research Institute, Nanchong Academy of Agricultural Sciences, Nanchong 637000, China

**Keywords:** potato, EPIDERMAL PATTERNING FACTOR, stomatal density, water-use efficiency, plant growth

## Abstract

Stomata are essential for photosynthesis and water-use efficiency in plants. When expressed in transgenic *Arabidopsis thaliana* plants, the potato (*Solanum tuberosum*) proteins EPIDERMAL PATTERNING FACTOR 2 (StEPF2) and StEPF-LIKE9 (StEPFL9) play antagonistic roles in regulating stomatal density. Little is known, however, about how these proteins regulate stomatal development, growth, and response to water deficit in potato. Transgenic potato plants overexpressing *StEPF2* (E2 plants) or *StEPFL9* (ST plants) were generated, and RT-PCR and Western blot analyses were used to select two lines overexpressing each gene. E2 plants showed reduced stomatal density, whereas ST plants produced excessive stomata. Under well-watered conditions, ST plants displayed vigorous growth with improved leaf gas exchange and also showed increased biomass/yields compared with non-transgenic and E2 plants. E2 plants maintained lower H_2_O_2_ content and higher levels of stomatal conductance and photosynthetic capacity than non-transgenic and ST plants, which resulted in higher water-use efficiency and biomass/yields during water restriction. These results suggest that StEPF2 and StEPFL9 functioned in pathways regulating stomatal development. These genes are thus promising candidates for use in future breeding programs aimed at increasing potato water-use efficiency and yield under climate change scenarios.

## 1. Introduction

Potato (*Solanum tuberosum* L.) is the third most consumed staple crop across the world. It is, however, a drought-susceptible plant that favors a cool climate, which makes it difficult to maintain yields given the increased occurrence of extreme weather events, including more and longer periods of low precipitation and high temperature; simultaneously, the growing world population is causing an increased demand for agricultural products [1]. These factors present grave challenges and require plant breeders to develop novel varieties of potatoes with improved stress resistance in response to the detrimental effects of climate change.

Stomata are microscopic pores in the epidermis of the aerial parts of plants that act to maximize gas diffusion, enabling carbon assimilation by photosynthesis, while minimizing water loss through transpiration [2]. Plants must therefore balance gas exchange with transpiration. They do so by altering the apertures of their stomata in response to various environmental cues, while also, over the long term, modifying the stomatal density in developing leaves [1,3]. Natural variation in the anatomical characteristics of stomata provides evidence of a potentially negative relationship between stomatal density and plant water-use efficiency (WUE) [4,5,6]. Furthermore, some plant species are able to maintain biomass yields at high temperatures due to increased stomatal density, which may aid transpiration and thus improve the process of cooling [7]. Therefore, altering the stomatal density is a potential method for developing crops that are resilient to climate change [8].

Stomatal development involves a series of cell fate transitions and differentiation processes that are regulated by a network of signaling pathways and regulatory factors, including basic helix–loop–helix (bHLH) transcription factors (TFs), the EPIDERMAL PATTERNING FACTOR (EPF)/EPF-LIKE (EPFL) ligand family, TOO MANY MOUTHS (TMMs), the ERECTA receptor complex, and the mitogen-activated protein kinase (MAPK) signaling cascade [9,10,11]. The ligands–receptors–MAPKs/TFs integrate intrinsic and extrinsic signals, forming a negative feedback loop that precisely controls stomatal development and distribution [12,13]. The EPF/EPFLs are a family of cysteine-rich intracellular signaling peptides that maintain the correct density and spacing of stomatal precursor cells by binding to ERECTA family receptors [14]. The *Arabidopsis thaliana* (Arabidopsis) genome contains, in total, 11 members of the EPFL family; three of these peptides, EPF1, EPF2, and EPFL9/STOMAGEN, play essential roles in stomatal development [15,16]. EPF1 and EPF2 are both secreted from stomatal lineage cells and negatively regulate stomatal density, while EPFL9 is derived from mesophyll cells and promotes stomatal development by competing with EPF2 for binding to the ERECTA receptor [16,17]. *EPFL* genes are conserved across many species of land plants, including both monocots and dicots, and C3 and C4 plants. Genetic manipulation or mutation of the expression patterns of EPF1, EPF2, and EPFL9 alters stomatal development, enabling the desired photosynthetic and transpiration traits to be obtained in Arabidopsis [18,19], rice (*Oryza sativa*) [20], wheat (*Triticum aestivum*) [21], maize (*Zea mays*) [22], and poplar (*Populus* spp.) [23]. Such studies encouraged us to explore whether the orthologous genes in potato have a similar function in the regulation of stomatal development in this crop species.

We reported previously that ectopic overexpression of potato *EPF2* (*StEPF2*) in Arabidopsis resulted in reduced stomatal density and enhanced drought tolerance, whereas plants overexpressing *StEPFL9* displayed the contrary phenotypes [24,25]. In the current study, we wished to determine if the StEPF2 and StEPFL9 function in the regulation of stomatal development was conserved in potato and to determine the impact of manipulating stomatal density on plant growth and drought tolerance. We therefore generated transgenic potato plants overexpressing either *StEPF2* or *StEPFL9* and investigated the effect of changes in the stomatal density on photosynthesis, drought tolerance, potato growth, and tuber yields.

## 2. Results

### 2.1. Generation of Transgenic Potato Plants

**Figure 1:**
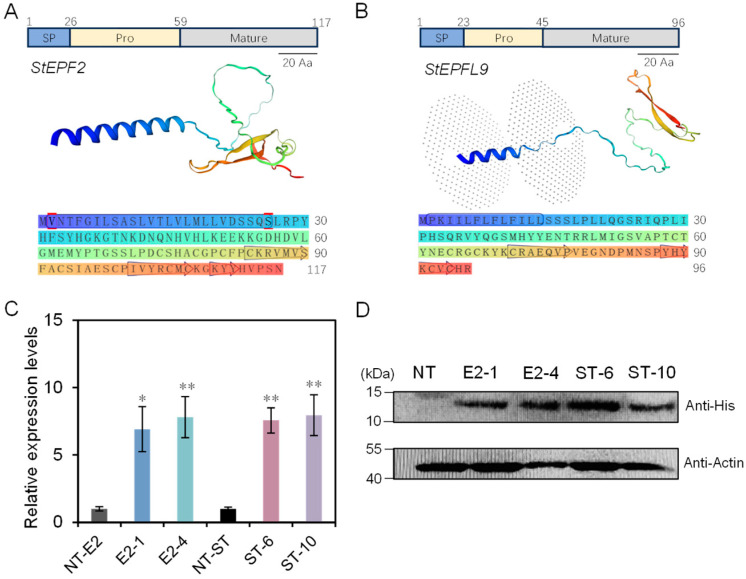
Bioinformatics analysis of the StEPF2 and StEPFL9 proteins. Structural models of (**A**) StEPF2 and (**B**) StEPFL9. SP: signal peptide; Pro: pro-peptide: Mature: mature peptide. (**C**) qRT-PCR analysis of *StEPF2* and *StEPFL9* expression; the potato gene *StEF1α* and *actin* were used as an internal control. (**D**) Western blot analysis of non-transgenic (NT) and transgenic plants. Data show the mean ± SE. Asterisks indicate significant differences between transgenic and NT lines by Duncan’s multiple range test; *: *p* < 0.05; **: *p* < 0.01.

As shown in Figure S1 and Figure 1A,B, StEPF2 and StEPFL9 proteins were predicted to be composed of three distinct parts: the N-terminal signal peptide, the pro-peptide domain, and the mature peptide, in which six cysteine residues formed a cyclic structure comprising three pairs of disulfide bonds. As shown in Figure S2, seven lines of transgenic plants overexpressing *StEPF2* (E2) and ten lines overexpressing *StEPFL9* (ST) were generated and we used genomic PCR analyses to confirm the presence of the transgene in each line. Subsequently, we selected four lines (E2-1, E2-4, ST-6, and ST-10) for further characterization by qRT-PCR and Western blot analyses (Figure 1C,D).

### 2.2. Characterization of Transgenic Potato Plants

**Figure 2:**
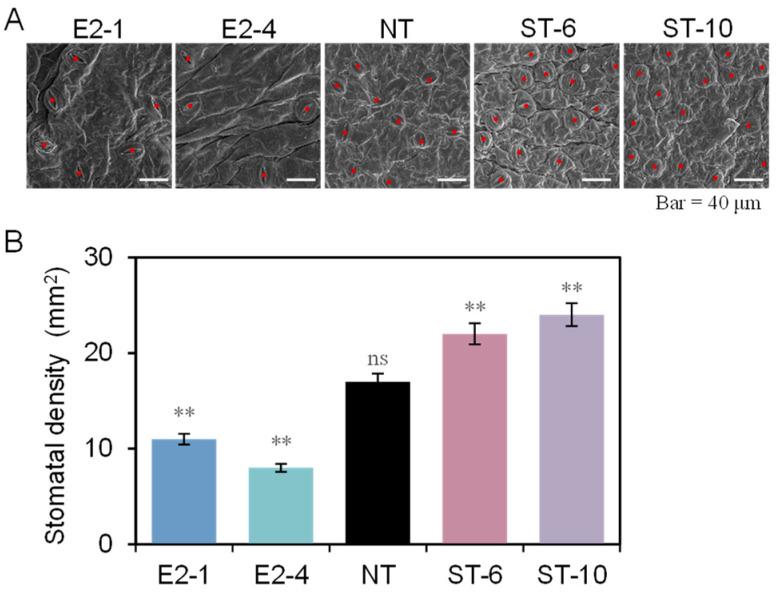
Phenotypic analyses of stomatal density in transgenic potato plants. (**A**) Photographs of the mature abaxial leaf epidermis of 3-week-old plants showing stomata. Scale bars: 40 μm. Red dots denote positions of stomatal complexes. (**B**) Stomatal density of non-transgenic (NT) and transgenic plants. Data show the mean ± SE. Asterisks indicate significant differences between transgenic and NT lines by Duncan’s multiple range test; ns: no significant difference; **: *p* < 0.01.

**Figure 3:**
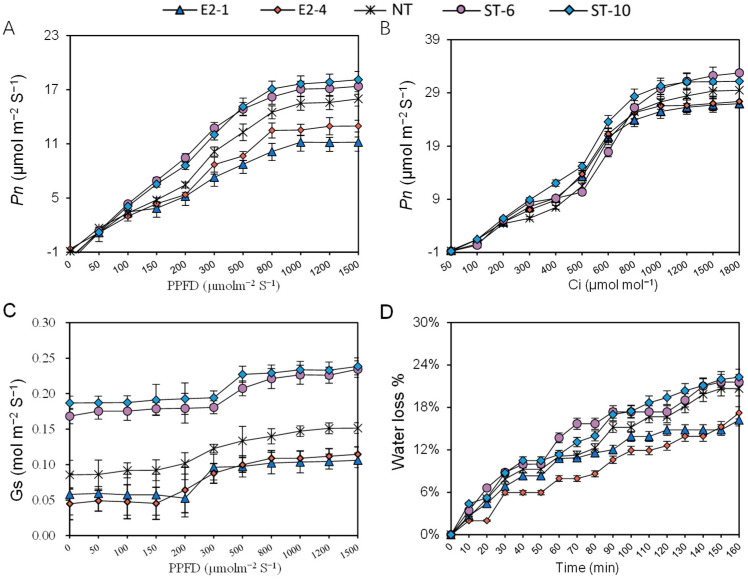
Photosynthetic responses of non-transgenic (NT) and transgenic potato plants. (**A**) *Pn-PPFD* curve. (**B**) *Pn-Ci* curve. (**C**) *Gs-PPFD* curve. (**D**) Water loss from detached leaves of 3-week-old NT and transgenic plants. Data show the mean ± SE. Leaves from the same position on each plant were used in this experiment.

As shown in Figure 2A, the stomatal density was reduced by 30.7% in the E2 lines, compared with NT plants, but stomatal density increased by 58.8% in the ST lines (Figure 2B), suggesting that StEPF2 and StEPL9 regulated stomatal density antagonistically in potato. As shown in Figure 3A–C, there was no significant difference in *Pn* under 100 μmol m^−2^s^−1^ or lower light conditions. The photosynthetic parameters, including *Pmax*, *Isat*, *Ic*, *Cisat*, *Amax*, and *Γ*, significantly increased in the ST lines, compared with NT plants, whereas they were significantly reduced in the E2 lines. Notably, the values of *CE* and *AQY* in the E2 lines did not differ significantly from those of NT plants, but were higher in the ST lines (Table S2). As shown in Figure 3D, the total water loss in the E2 lines was reduced by 3.5%, compared with NT plants, whereas it increased by 2.15% in the ST lines.

### 2.3. Relationship between Stomatal Density and Drought Tolerance in Transgenic Potato Plants

**Figure 4:**
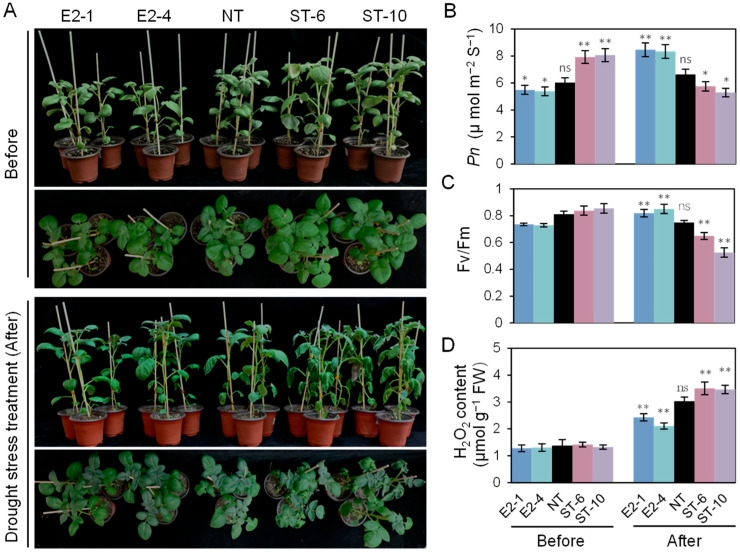
Phenotypic analyses of non-transgenic (NT) and transgenic potato plants under short-term drought stress conditions. (**A**) Appearance of plants before and after drought treatment. (**B**) *Pn*, (**C**) Fv/Fm, and (**D**) H_2_O_2_ content in non-transgenic (NT) and transgenic plants. Leaves from the same position on each plant were used in this experiment. Data show the mean ± SE. Asterisks indicate significant differences between transgenic and NT lines by Duncan’s multiple range test; ns: no significant difference; *: *p* < 0.05; **: *p* < 0.01.

As shown in Figure 4A–C, prior to drought treatment, increased levels of *Pn* and Fv/Fm were observed in the ST lines, but not in the E2 lines, compared with NT. Under persistent drought conditions, the leaves of the ST lines exhibited obvious wilting; the values of *Pn* and Fv/Fm in the ST lines decreased significantly compared with NT plants, whereas the values of *Pn* and Fv/Fm increased by 13.4% and 31.9%, respectively, in the E2 lines (Figure 4B,C). The H_2_O_2_ content in detached leaves of the ST lines was 34.5% higher than in NT plants, whereas it decreased by 22% in the E2 lines, which also accumulated lower levels of H_2_O_2_, visualized by 3,3′-diaminobenzidine (DAB) staining (Figure 4D and Figure S3).

### 2.4. Growth of Transgenic Potato Plants under Long-Term Drought Stress

**Figure 5:**
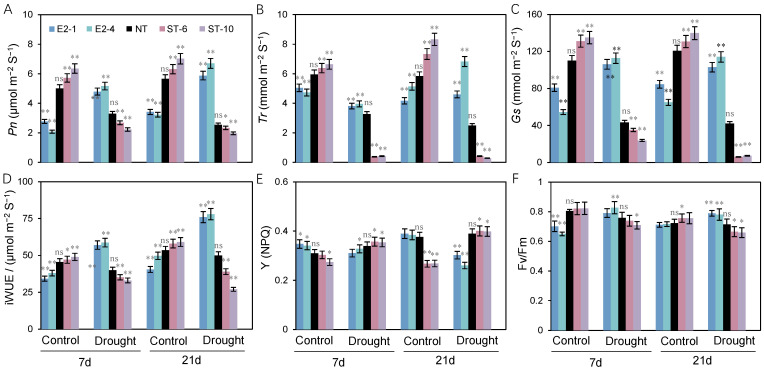
Photosynthetic and chlorophyll fluorescence parameters of transgenic potato plants under long-term drought stress. (**A**) *Pn*, (**B**) *Tr*, (**C**) *Gs*, (**D**) iWUE, (**E**) Y(NPQ), and (**F**) Fv/Fm in non-transgenic (NT) and transgenic plants after 7 or 21 days under well-watered and water-restricted conditions. Leaves from the same position on each plant were used in this experiment. Data show the mean ± SE. Asterisks indicate transgenic and NT lines differed significantly by Duncan’s multiple range test; ns: no significant difference; *: *p* < 0.05; **: *p* < 0.01.

As shown in Figure 5, when compared with NT plants, the values of *Pn*, *Gs*, *Tr*, iWUE, and Fv/Fm in the ST lines in the well-watered condition increased significantly by 26.7%, 22.6%, 11.4%, 7.6%, and 2.1%, respectively; by contrast, the values of these parameters decreased by 7.5%, 36.2%, 18.0%, 4.7%, and 1.3%, respectively, in well-watered plants from the E2 lines. Y(NPQ) increased by 1.2% in the E2 lines but decreased in the ST lines, relative to NT plants (Figure 5E). Under water-restricted conditions, the values of *Pn*, *Gs*, *Tr*, iWUE, and Fv/Fm in the ST lines showed 1.09-, 6.20-, 1.28-, 2.20-, and 1.07-fold decreases, respectively, compared with NT plants, whereas these parameters showed 2.60-, 2.70-, 2.10-, 1.56-, and 1.10-fold increases, respectively, in the E2 lines. Conversely, the ST lines exhibited a significant increase (5%) in Y(NPQ), compared with NT plants, while the E2 lines showed a 10.2% reduction (Figure 5).

**Figure 6:**
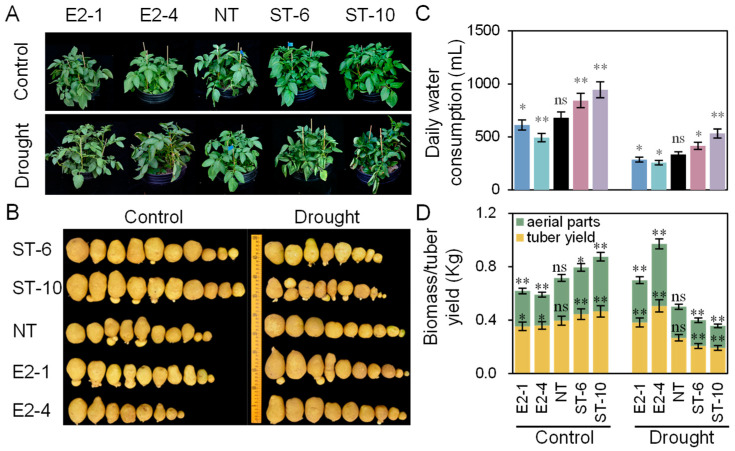
Effect of long-term drought treatment on production of NT and transgenic potato plants. (**A**) Phenotypes of 2-month-old aerial parts of NT, E2, and ST plants under well-watered and water-restricted conditions. (**B**) Phenotypes of tubers produced by NT and transgenic potato plants after harvest. (**C**) Daily water consumption of each line during drought treatment. (**D**) Biomass/tuber yield of NT and transgenic potato plants. Data show the mean ± SE, and each sample (three plants per pot) was replicated five times. Asterisks indicate transgenic and NT lines differed significantly by Duncan’s multiple range test; ns: no significant difference; *: *p* < 0.05; **: *p* < 0.01.

Under well-watered conditions, the ST lines grew more vigorously than the NT and E2 plants (Figure 6A), and the daily water consumption, biomass, and yield of the ST lines increased by 23.6%, 9.7%, and 20.6%, compared with these of NT plants, but decreased by 10.2%, 7.1%, and 9.7% in the E2 lines, respectively (Figure 6B–D). Conversely, under water-restricted conditions, compared with NT plants, the biomass and yield of the E2 lines increased by 19.9% and 32.9%, but decreased by 14.2 and 28.3% in the ST lines, respectively (Figure 6B,D). Interestingly, under water-restricted conditions, the daily water consumption of the ST lines increased by 33.8% over that of NT plants, whereas that of the E2 lines decreased by 22.7% (Figure 6C).

## 3. Discussion

Stomata play a vital role in ensuring plant survival by regulating gas exchange, water loss, and pathogen entry [26]. Our knowledge of the networks of genes implicated in potato stomatal development remains limited, however. In the current study, genetic manipulation of stomatal density was achieved via regulating two EPF orthologues in potato (StEPF2 and StEPFL9) to investigate the impact of stomatal density on potato growth and water-use efficiency.

Several lines of evidence indicate that StEPF2 and StEPFL9 are functional orthologues of EPF2 and EPFL9 from other plants. Mature EPF proteins possess a distinctive knotted structure scaffold, in which three disulfide bonds form between a set of conserved cysteine (C) residues. A variable loop region between C4 and C5 determines the antagonistic activity between EPF2 and EPFL9 [27]. Our study found that the amino acid sequences of StEPF2 and StEPFL9 were highly conserved and closely resembled those from other species. The proteins contained a signal peptide at the N terminus, a pro-peptide domain, and the amino acid residues of the mature protein. Stomatal density was decreased in potato plants overexpressing *StEPF2* and increased in potato plants overexpressing *StEPFL9*, consistent with previous studies of *EPF2* and *EPFL9* orthologues in other plants. Studies manipulating the expression of the *EPF2* and *EPFl9* genes in a range of plant species, including *AtEPF2* (Arabidopsis) [18], *PdEPF2* (white poplar) [28], *OsEPF2* (rice) [20], *SbEPF2* (sorghum) [29], *TaEPF2* (bread wheat) [30], *MdEPF2* (apple) [31], *BnaEPF2* (Brassica napus) [32], *AtEPFL9* [19], *OsEPFL9* [20], and *VvEPFL9* (grapevine) [33], have shown their products, EPF2 and EPFL9, play antagonistic roles in modulating stomatal development. These results provide strong evidence that the *EPF* genes in potato were, at least in part, highly conserved, both structurally and functionally.

It is predicted that, due to climate change, extreme weather events, such as drought and high temperature, will occur at an increasingly high frequency; this will seriously affect the growth and yield of future crops [34]. Stomata are the main facilitators of transpiration as, typically 97% of absorbed water escapes though stomata to the atmosphere, but they are also essential for the uptake of the atmospheric CO_2_ used in mesophyll photosynthesis [26]. Plants with lower stomatal density are usually more drought-tolerant and show a higher efficiency in water use under water-deficit conditions [35]. We found that potato plants with reduced stomatal density maintained relative high rates of photosynthesis (*Pn*), transpiration (*Tr*), stomatal conductance (*Gs*), and intrinsic WUE (iWUE) under drought stress treatment. Similarly, genetic manipulation or mutations that reduced stomatal density improve WUE and drought tolerance in several monocot species, including rice [36], sorghum [29], barley [37], and wheat [30], as well as in dicot species such as Arabidopsis [5], poplar [38], grapevine [33], tomato (*Solanum lycopersicum*) [31], and B. napus [32]. Taken together, these findings suggest that reducing the stomatal density of leaves, either by overexpressing *EPF2* or downregulating the expression of *EPFL9*, was an effective strategy for improving drought tolerance and WUE in plants. Conversely, plants with increased stomatal density were likely to exhibit enhanced Gs, carbon dioxide assimilation, and *Pn*. Overexpression of *EPFL9* in Arabidopsis [39], rice [20], poplar [40], and potato (this study), as well as disruption of the Arabidopsis *EPF2* orthologue, increased stomatal density and also enhanced *Pn*, *Gs*, and *Tr* under well-watered conditions. The carboxylation capacity did not differ between *AtEPFL9* overexpression lines and wild-type Arabidopsis plants, which suggested that increasing stomatal density altered photosynthetic capacity by modulating CO_2_ diffusion rather than Rubisco activity [39]. The rate of water loss in detached leaves of E2 plants was higher than that in NT and E2 plants, in which lower water potential might have caused stomatal closure. The E2 plants showed consistently lower *Tr* and *Gs* under water-restricted conditions and were much more susceptible to wilting than NT and E2 plants.

Although decreasing stomatal density is an efficient strategy for improving plant tolerance to drought, a reduction in stomatal conductance may affect the rate of CO_2_ assimilation, thus reducing biomass production due to the restriction on gas exchange [35]. Investigations in several grass species, however, indicate that a reduction in stomatal density may improve both WUE and drought tolerance without having a negative impact on yields. Overexpression of *HvEPF1* in barley [37], of *OsEPF1* in rice [36], and of *TaEPF2* in bread wheat [30] increases plant resistance to reduced water availability without affecting yield, when the reduction in the number of stomata is moderate. In rice, only lines with a marked reduction (88%) in stomatal densities show a statistically significant decrease in CO_2_ assimilation [36]; similarly, wheat lines with >50% reductions in stomatal density were highly susceptible to yield losses [30]. In the current study, the biomass and tuber yield were reduced under well-watered conditions in potato plants with decreased stomatal density, but biomass production and tuber yield were relatively high when the plants were exposed to water-restricted conditions. Several studies that compared stomatal opening and closing responses between grass species with subsidiary cells and those with kidney-shaped stomata suggest that grasses exhibit faster and more efficient stomatal regulation than other plants; this is likely to improve WUE and reduce the risk of disruption to the leaf hydraulic system in grass species [35]. E2 potato plants displayed an enhanced rate of plant growth under optimal growth conditions; this was consistent with a previous study of transgenic poplar that showed plants overexpressing *PagEPFL9* exhibit increased plant height and biomass [40]. It is possible that high stomatal density increases the potential rate of gas exchange, which would have beneficial effects on CO_2_ assimilation and also ensure a relatively stable leaf temperature by enabling evaporative cooling to dissipate heat at times of high temperature.

## 4. Materials and Methods

### 4.1. Plant Materials and Growth Conditions

All potato plants used in this study were of the Atlantic cultivar (*Solanum tuberosum* L. cv. Atlantic, which is model cultivar for potato transformation and functional analysis of genes and a drought-tolerant cultivar [41]). Plants were sub-cultured monthly by transferring stem cuttings with double nodes to fresh rooting medium (RM; i.e., 1 × Murashige and Skoog (MS) medium). Two groups of experiments were conducted. For short-term measurements of drought stress, following culture on RM for one-month, fifteen rooted plantlets for each line were transplanted into each pot (8 × 9 × 5.5 cm) filled with sterile matrix, vermiculite, and perlite (3:1:1) and placed in a growth chamber at 25 °C, 60% relative humidity, and a 16/8 h (light/dark) photoperiod with a light intensity of 100 μmol m^−2^ s^−1^.

For long-term drought treatments, fifteen rooted potato plants for each line were transplanted into plastic pots (30 cm diameter × 29 cm height; three seedlings per pot) after a 7-day acclimatization period, cultivated at Yangling (34°17′00″ N, 108°03′42″ E), China. The highest temperature during the period of cultivation was 27 °C, and the lowest was 12.75 °C; shelters were used to keep out the rain. The soil was calcic cambisol [42], pH 8.21, with an organic matter content of 2.26 g kg^−1^, and total nitrogen (N), phosphorus (P), and potassium (K) contents were 0.22, 0.54, and 0.23 g kg^−1^, respectively. N, P, and K (0.25, 0.17, and 0.23 g kg^−1^ dried soil) were applied as base fertilizers. Pot weight was measured on a daily basis to determine the percentage of the initial soil water content remaining, as well as water consumption. The soil water content was maintained at 80% in the well-watered control group; fifteen potato plants in tuber initiation stage were exposed to drought treatment; the relative soil water content was allowed to fall progressively for the first 7–8 days and then maintained at approximately 40% until harvest using a daily water supplement (Figure S4).

### 4.2. Bioinformatics Analysis

BLAST searches were conducted using the GenBank database to identify the nucleotide sequences of EPF2/EPFL9 family members. The online programs ClustalW and ESPript 3.0 (https://espript.ibcp.fr, accessed on 6 June 2024) were used to perform multiple sequence alignments (Figure S1). SignaIP 4.1 (http://www.cbs.dtu.dk/services/signalp/, accessed on 6 June 2024) was used to predict and analyze the signal peptides. The three-dimensional structures of the proteins were simulated using SWISS MODEL (https://swissmodel.expasy.org/interactive, accessed on 6 June 2024).

### 4.3. Potato Transformation

To investigate the function of StEPF2 and StEPFL9 in regulating stomatal density in potato plants, the construct pCAMBIA1380-35S:StEPF2/StEPFL9 was introduced into potato plants using *Agrobacterium tumefaciens* (strain EHA105)-mediated transformation, as described by Ahmad et al. (2008) [43]. The stem internodes from thirty 3-week-old cultured potato seedlings for each gene were transformed; explants were pre-cultured in a culture dish for 2 days and then inoculated with *A. tumefaciens* for 15 min. After incubation for 2 days on co-culture medium, comprising 1 × MS medium containing 2.0 mg/L 2,4-D, the explants were transferred to shoot induction medium containing 2.0 mg/L zeatin, 0.1 mg/L GA3, 0.01 mg/L NAA, and 5 mg/L hygromycin. Hygromycin-resistant shoots were transferred to fresh RM for rooting. Transformation of the rooted plantlets was confirmed using PCR.

### 4.4. Gene Expression in Potato Plants

The PhirePlant Direct PCR kit (Thermo Scientific, Waltham, MA, USA) was used to analyze genomic DNA. For quantitative real-time (qRT)-PCR analyses, total RNA was extracted from six 3-week-old potato plants using TRIzol reagent and reverse-transcribed using the PrimeScript II 1st strand cDNA synthesis kit (Takara, Beijing, China). qRT-PCR was performed using a QuantStudio™ 5 Real-Time PCR System with SYBR Premix Ex Taq Kit (Takara, Beijing, China) and the gene-specific primers listed in Table S1. The relative level of expression of each gene of interest was quantified using the 2^−ΔΔCT^ method with *StEF1a* and *actin* as the internal reference genes [44]. All reactions were performed in triplicate using three independent cDNA samples.

### 4.5. Protein Immunoblotting

Total protein was extracted from six 3-week-old potato plants grown in pots. Approximately 0.2 g of ground leaf samples was combined with extraction solution containing 50 mM Tris-HCl (pH 7.5), 5 mM EDTA, 5 mM EGTA, 10 mM DTT, and one protease inhibitor tablet (Roche, Mannheim, Germany). Proteins were separated by SDS-PAGE and electro-transferred onto a polyvinylidene fluoride membrane. Membranes were blocked in TBST buffer with 6% skimmed milk at 4 °C overnight. For the immunoblot analyses, we used anti-Actin and anti-His as the primary antibodies and anti-IgG as the secondary antibody (Sangon, Shanghai, China).

### 4.6. Stomatal Density

To assess the effects of overexpression of *StEPF2* and *StEPFL9* on stomatal density, stomata on the abaxial leaf epidermis of transgenic potato plants were observed using a scanning electron microscope. Briefly, leaf (3rd fully expanded leaves from the top) samples from 3-week-old potato plants were fixed in 4% glutaraldehyde in 0.1 M phosphate buffer (pH 7.2) for 4 h and dehydrated across a gradient of 30% to 100% ethanol; samples were placed in each concentration for 15 min. Finally, samples were moved through three changes of 100% tert-butanol. Samples were oven-dried at 40 °C overnight and coated with a thin layer of gold prior to scanning electron microscopy analysis (TCS SP8, LEICA, Germany). Eight leaf discs (two leaf discs per leaf) per potato line were measured. There were three biological repeats per data point.

### 4.7. Leaf Gas Exchange and Chlorophyll Fluorescence Measurements

To determine whether the changes in stomatal density affected gas exchange, we measured the Pn-PPFD, Gs-PPFD, and Pn-Ci response curves in fully expanded leaves. Briefly, gas exchange was measured on sunny days in 3rd fully expanded leaves (from the top) from 3-week-old plants using a Li-6800 portable gas exchange system (LI-COR, Lincoln, NE, USA). Leaves located at the same position on each plant were used in this experiment. The environmental conditions within the leaf chamber were light intensity of PPFD = 1000 μmol m^−2^s^−1^, 50% humidity, 25 °C leaf temperature, 500 μmol s^−1^ airflow, and 400 ppm CO_2_. The light intensity gradients were 1500, 1000, 800, 600, 400, 300, 200, 150, 100, 50, and 0 μmol m^−2^ s^−1^. The net photosynthetic rate (*Pn*), stomatal conductance rate (*Gs*), and transpiration rate (*Tr*) were measured simultaneously. The instantaneous WUE (iWUE) of the leaf was calculated as the rate of *Pn* to *Tr*.

The *Pn-Ci* response curve was generated by measuring the photosynthetic rate in leaves exposed to 400, 200, 100, 50, 300, 500, 600, 800, 1000, 1200, 1500, and 1800 ppm CO_2_. The modified rectangular hyperbola model was used to match the Pn-Ci response curve and to calculate the initial carboxylation efficiency (*CE*), photosynthetic capacity (*Amax*), saturated intercellular CO_2_ concentration (*Cisat*), CO_2_ compensation point (*Γ*), rate of photorespiration (*Rp*), apparent quantum efficiency (*AQY*), light saturation point (*Isat*), dark respiration rate (*Rd*), and light compensation point (*Ic*). The quantum efficiency of photosystem II (PSII) was determined in expanded leaves after dark adaptation for 30 min by calculating the ratio of variable fluorescence to maximum fluorescence (Fv/Fm), and non-photochemical quenching (NPQ) of leaves was measured using the MINI-PAM-II chlorophyll fluorescence system (Walz, Effeltrich, Germany). All experiments were three biological repeats with five leaves in each replicate.

### 4.8. Quantification and Detection of Hydrogen Peroxide (H_2_O_2_)

The 3rd leaves from the top were collected from 3-week-old potato plants cultured in pots and ground in liquid nitrogen. H_2_O_2_ levels were measured using the Hydrogen Peroxide Assay Kit (Comin, Nanjing, China), according to the manufacturer’s protocol. There were five biological repeats with three leaves in each replicate.

### 4.9. Measurement of Water Loss from Detached Leaves

To assess the effect of the alterations in stomatal density on water loss, water loss was measured in detached leaves (3rd leaves from the top) from 3-week-old potted seedlings. Nail polish was applied to the stem cross-section to prevent transpiration. The weight of the detached leaves was measured at 10 min intervals; water loss between 0 and 3 h was calculated using (initial weight–test weight)/initial weight × 100%; this process was repeated five times for each group of leaves with three leaves in each replicate.

### 4.10. Measurement of Daily Water Consumption, Biomass, and Tuber Yield

To assess the changes in yield of transgenic potato plants under long-term drought stress conditions, we evaluated the biomass and tuber yields and calculated the mean daily water consumption following a period of drought treatment. Briefly, the daily water consumption of NT and transgenic lines was equivalent to the average amount of water supplement per pot during the drought treatment. The growth cycle of potato plants lasted 84 days; the aerial parts and tubers were harvested, and their fresh weights were immediately measured. Each sample (three plants per pot) was replicated five times.

### 4.11. Statistical Analyses

Statistical analyses were performed using the SPSS version 26.0 (SPSS, Chicago, IL, USA). One-way ANOVA with Duncan’s multiple comparison was used to test for differences between lines of plants. All values are presented as means ± standard error (SE). Statistically significant differences between means of non-transgenic (NT) and transgenic plants are indicated by asterisks: *: *p* < 0.05; **: *p* < 0.01.

## 5. Conclusions

To conclude, our results show that StEPF2 and StEPFL9 play opposing roles in regulating stomatal development and potato plants with reduced stomatal density improved drought tolerance under deficit conditions, while potato plants with increased stomatal density enhanced plant growth and tuber yields under well-watered conditions. This study increases our understanding of the complex regulatory mechanism underlying stomatal development and demonstrates the correlations among stomatal density, plant growth, and drought tolerance in potato. The potato plants developed in this study must be further evaluated in the field under naturally fluctuating environmental conditions. Furthermore, despite the restrictions on the cultivation of GM plants imposed by many countries, stomatal development can be modified via exogenous application of synthetic EPF peptides. In future, the manipulation of stomatal density may be a useful way for plant breeders to optimize potato yield in face of the challenge posed by climate change.

## Data Availability

No large datasets were created in this study.

## Supplementary Materials

The following supporting information can be downloaded at https://www.mdpi.com/article/10.3390/ijms251910738/s1.
