# Supplementary material for: StEPF2 and StEPFL9 Play Opposing Roles in Regulating Stomatal Development and Drought Tolerance in Potato (Solanum tuberosum L.)
Source: Int J Mol Sci. 2024 Oct 5;25(19):10738. doi: 10.3390/ijms251910738 (PMC11476617; doi:10.3390/ijms251910738)
Supplement: Supplementary file 1 [file ijms-25-10738-s001.zip › Supplementary_Figure.pdf]

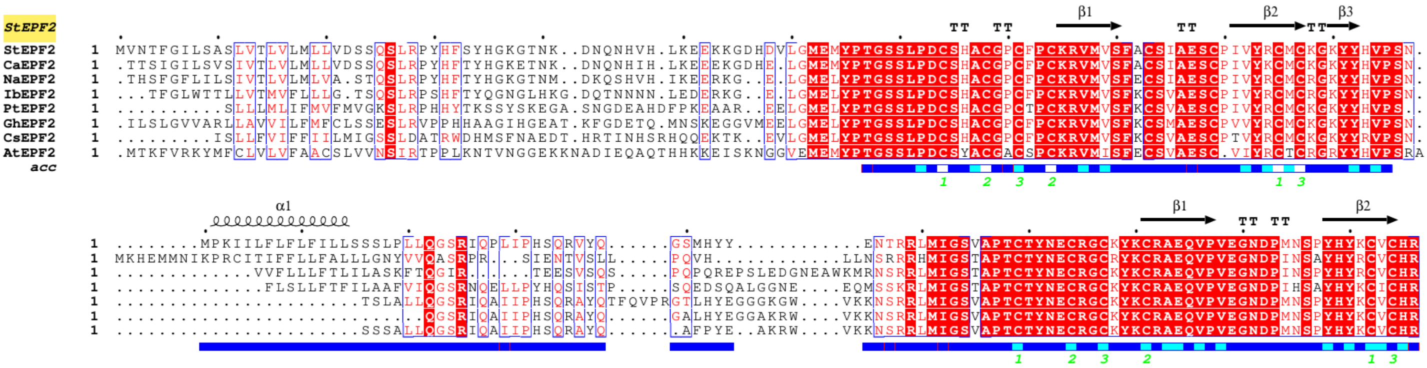

Figure S1. Alignment of amino acid sequences from StEPF2 and StEPFL9 with protein sequences from other species. Cysteine residues are labeled in green; strictly conserved amino acid residues are labeled in red; solvent accessibility scales (acc) per residue are shown in blue.

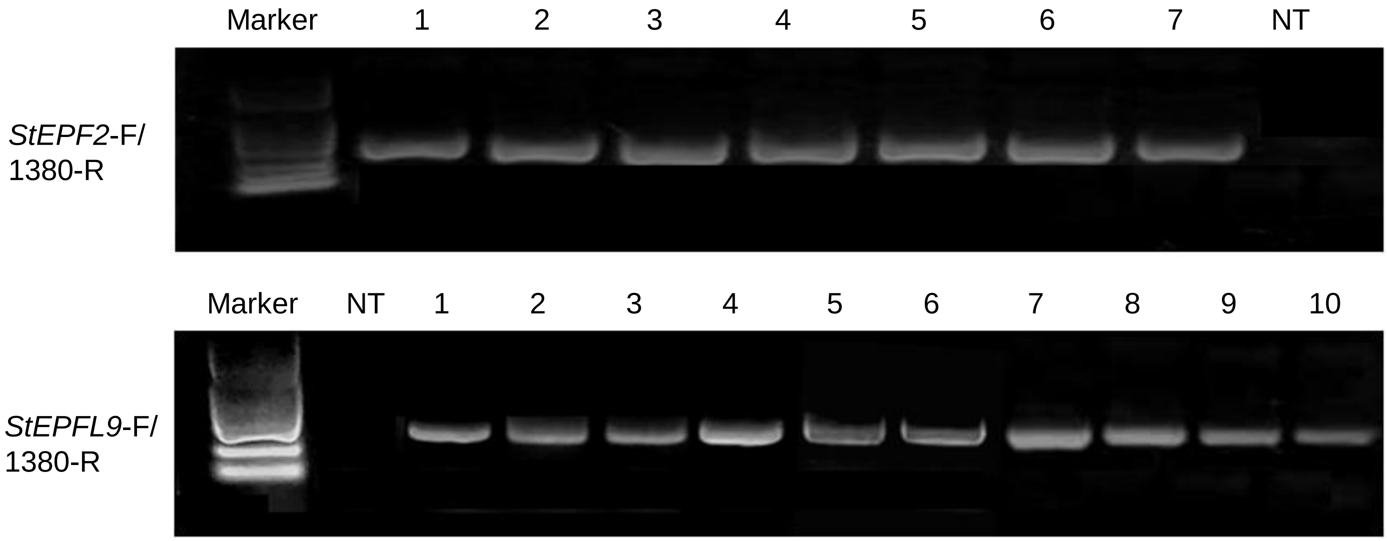

Figure S2. PCR analyses of genomic DNA from the E2 and ST plants.

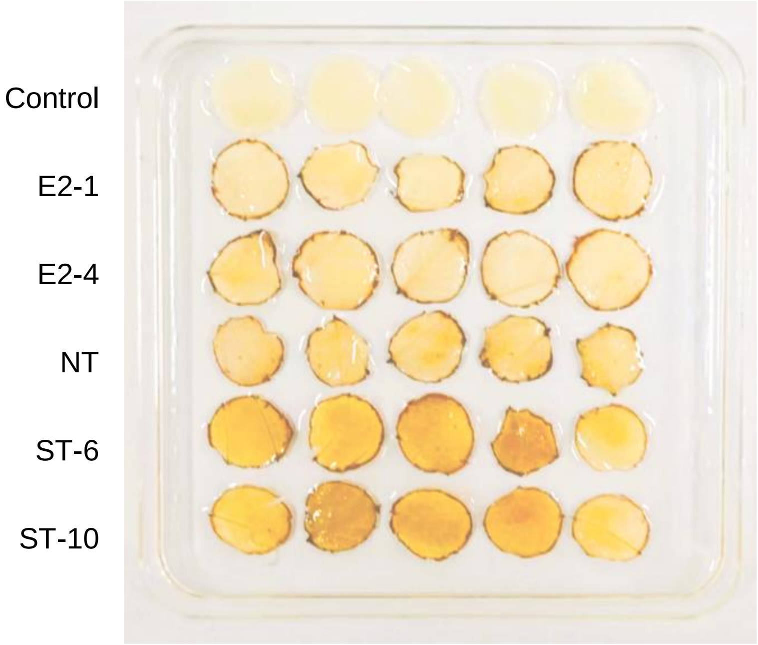

Figure S3 3, 3'-diaminobenzidine (DAB) staining of detached leaves of E2 and ST plants to show reactive oxygen species (ROS) accumulation.

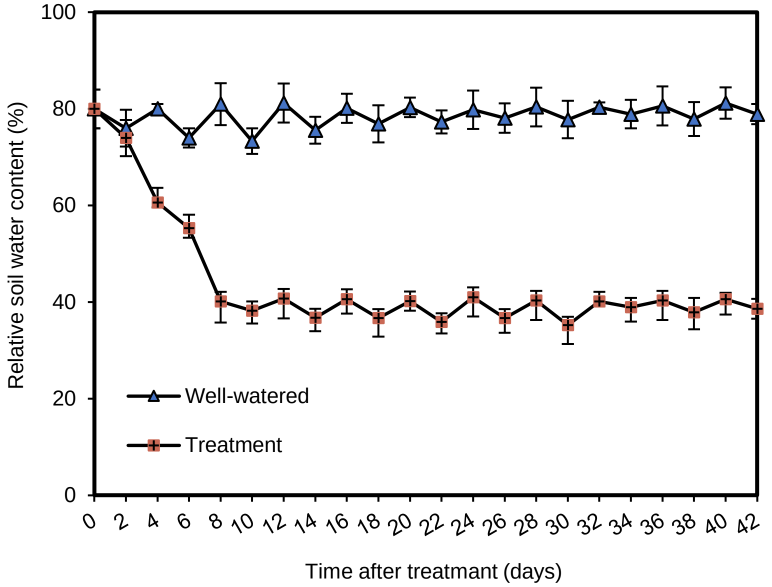

Figure S4. Soil water content under drought stress conditions. Data show the mean ± standard error (SE).
