# Supplementary material for: StEPF2 and StEPFL9 Play Opposing Roles in Regulating Stomatal Development and Drought Tolerance in Potato (Solanum tuberosum L.)
Source: Int J Mol Sci. 2024 Oct 5;25(19):10738. doi: 10.3390/ijms251910738 (PMC11476617; doi:10.3390/ijms251910738)
Supplement: Supplementary file 1 [file ijms-25-10738-s001.zip › table_S1.pdf]

Table S1. Primers used in this study.

Primer name	Primer sequence(5'-···-3')	Purpose of primers
StEPF2 – F	ACTAGTATGGTGAATACATTTGGA	Amplify the target gene
StEPF2 – R	GGATCCTGTCCCTTCCAATTGA	Amplify the target gene
StEPFL9 – F	ACTAGTATGACGAGAGGTGAGGAAG	Amplify the target gene
StEPFL9 – R	GGATCCAGTTGATTCATTGATCAAG	Amplify the target gene
1380-R	GTGTCGTGCTCCACCATG	Amplify the target gene
StEPF2 – RT – F	TCTAGCCAAAGCCTACGTCC	qRT – PCR
StEPF2 – RT – R	GGGAAACAAGGTCCACAAGC	qRT – PCR
StEPFL9 – RT – F	TCAGGGTTCGATGCACTACT	qRT – PCR
StEPFL9 – RT – R	CAACTGGAACTTGCTCTGCTC	qRT – PCR
Stef1α – RT – F	CTGGTACAAGGGACCAACCC	Reference gene
Stef1α – RT – R	ACACCAGTCTCAACACGACC	Reference gene
actin-RT-F	GCTTCCCGATGGTCAAGTCA	Reference gene
actin-RT-R	GGATTCCAGCTGCTTCCATTC	Reference gene
