# Supplementary material for: StEPF2 and StEPFL9 Play Opposing Roles in Regulating Stomatal Development and Drought Tolerance in Potato (Solanum tuberosum L.)
Source: Int J Mol Sci. 2024 Oct 5;25(19):10738. doi: 10.3390/ijms251910738 (PMC11476617; doi:10.3390/ijms251910738)
Supplement: Supplementary file 1 [file ijms-25-10738-s001.zip › table_S2.pdf]

Table S2. Photosynthetic parameters based on modified rectangular hyperbola model.

	CE (mol m^{-2} s^{-1})	*Amax* (μmol m^{-2} s^{-1})	*Cisat* (μmol/mol)	*Γ* (μmol/mol)	*Rp* (μmol m-^{2} s^{-1})
E2-1	0.0453±0.00227a	28.0497±0.84149b	1534.7912±46.04374c	108.0367±3.2411c	3.9691±0.11907b
E2-4	0.0470±0.00335a	28.7338±0.86201b	1638.7646±49.16294d	103.0005±3.09002b	4.2995±0.12899b
NT	0.0460±0.00234a	30.5009±0.91503a	1441.0967±43.2329a	87.2285±2.61686a	3.5477±0.10643a
ST-6	0.0475±0.00213a	33.4666±1.00400c	1417.7382±42.53215b	80.7467±2.4224d	4.7941±0.14382c
ST-10	0.0536±0.00268b	32.8450±0.98535bc	1417.5884±42.52765b	82.7612±2.48284d	4.6396±0.13919c

	AQY (α)	*Pmax* (μmol m^{-2} s^{-1})	*Isat* (μmol m^{-2} s^{-1})	*Ic* (μmol m^{-2} s^{-1})	*Rd* (μmol m^{-2} s^{-1})
E2-1	0.0725±0.00363a	9.5372±0.28612b	1273.8791±38.21637c	17.5079±0.52524c	1.5536±0.04661c
E2-4	0.071±0.00355a	9.246±0.27738b	1337.6903±40.13071c	17.1589±0.51477c	1.7886±0.05366c
NT	0.0727±0.00354a	10.7533±0.3226a	1642.575±49.27725a	22.9732±0.6892a	1.1245±0.03374a
ST-6	0.1093±0.00547b	14.3987±0.4319c	1857.7325±55.73198b	26.8658±0.80597b	1.1471±0.03441b
ST-10	0.1177±0.00589b	17.8982±0.53695c	1945.6723±58.37017b	26.88±0.8064b	1.7524±0.05257c

CE: initial carboxylation efficiency; *Amax*: photosynthetic capacity; *Cisat*: saturated intercellular CO2 concentration; *Γ*: CO2 compensation point; *Rp*: rate of photorespiration; AQY: apparent quantum efficiency; *Isat*: light saturation point; *Rd*: dark respiration rate; *Ic*: light compensation point. Data are the mean ± SE; each value is the mean of four replicates; different alphabets indicate significance at $P < 0.05$.
