# Supplementary figures and images for: StEPF2 and StEPFL9 Play Opposing Roles in Regulating Stomatal Development and Drought Tolerance in Potato (Solanum tuberosum L.)
Source: Int J Mol Sci. 2024 Oct 5;25(19):10738. doi: 10.3390/ijms251910738 (PMC11476617; doi:10.3390/ijms251910738)

IJMS-3193409 – Original Blots

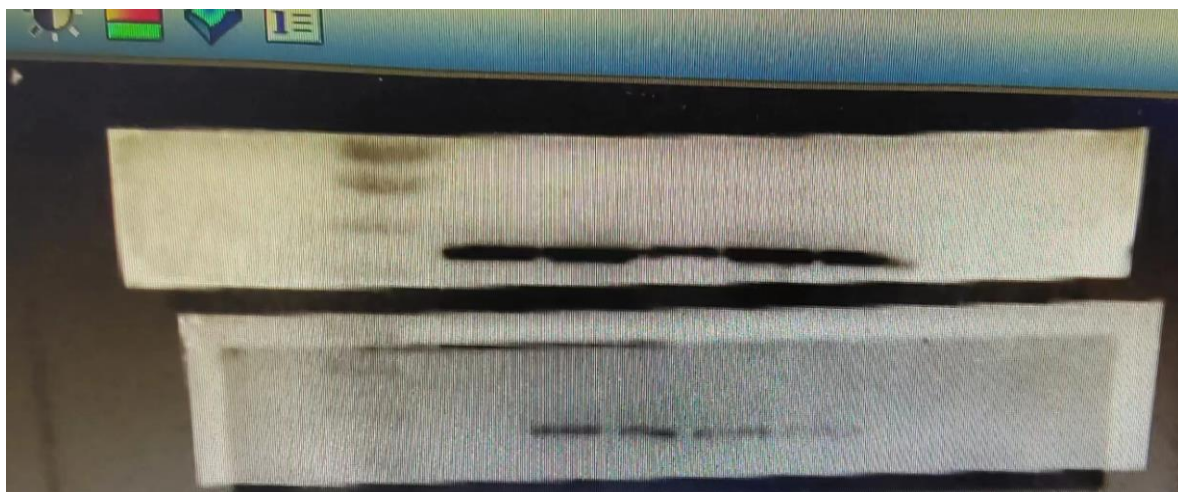

Figure 1D: Anti-Actin (Upper Part)

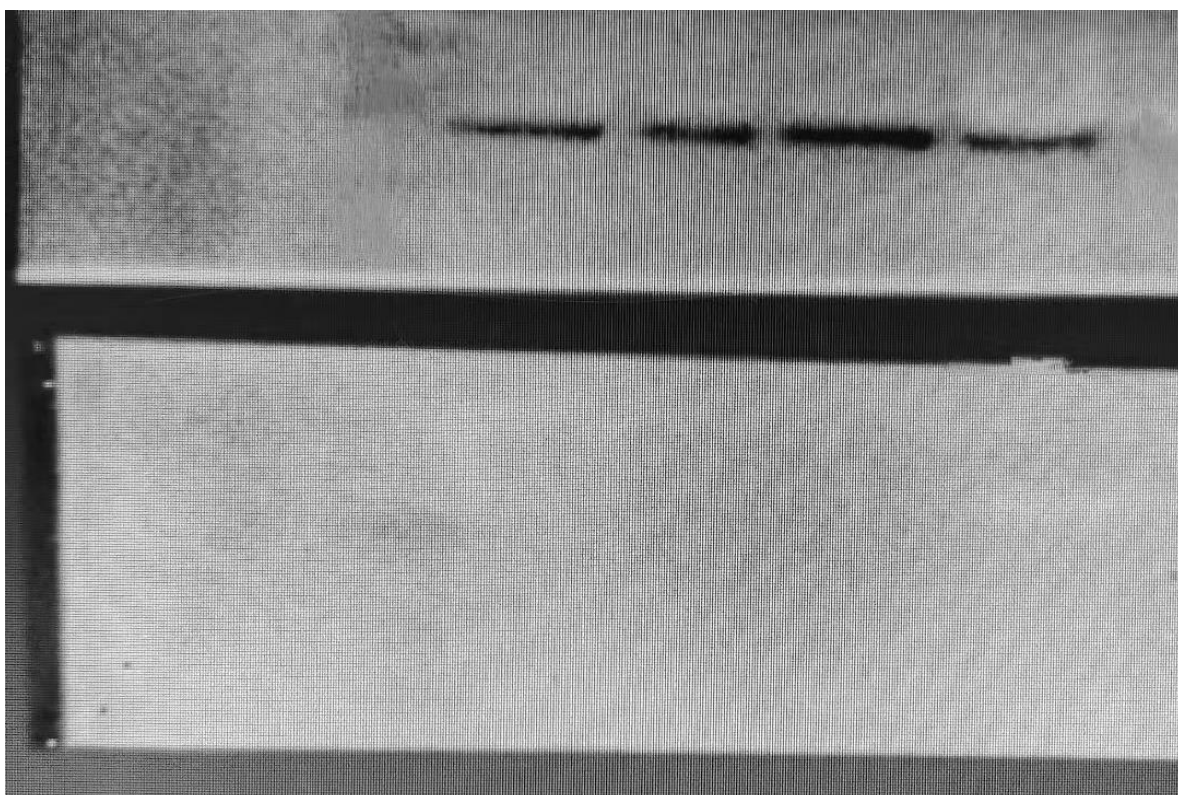

Figure 1D: Anti-His (Upper Part)

Supplement: Supplementary file 1 [file ijms-25-10738-s001.zip › IJMS-3193409 – Original Blots.pdf]
